# iApp: An Autonomous Inspection, Auscultation, Percussion, and Palpation Platform

**DOI:** 10.3389/fphys.2022.825612

**Published:** 2022-02-14

**Authors:** Semin Ryu, Seung-Chan Kim, Dong-Ok Won, Chang Seok Bang, Jeong-Hwan Koh, In cheol Jeong

**Affiliations:** ^1^School of Artificial Intelligence Convergence, Hallym University, Chuncheon, South Korea; ^2^Department of Sport Interaction Science, Sungkyunkwan University, Suwon, South Korea; ^3^Department of Internal Medicine, Hallym University College of Medicine, Chuncheon, South Korea; ^4^Department of Population Health Science and Policy, Icahn School of Medicine at Mount Sinai, New York, NY, United States

**Keywords:** remote patient monitoring, deep learning, clinical decision support systems, inspection, auscultation, percussion, palpation

## Abstract

Disease symptoms often contain features that are not routinely recognized by patients but can be identified through indirect inspection or diagnosis by medical professionals. Telemedicine requires sufficient information for aiding doctors' diagnosis, and it has been primarily achieved by clinical decision support systems (CDSSs) utilizing visual information. However, additional medical diagnostic tools are needed for improving CDSSs. Moreover, since the COVID-19 pandemic, telemedicine has garnered increasing attention, and basic diagnostic tools (e.g., classical examination) have become the most important components of a comprehensive framework. This study proposes a conceptual system, iApp, that can collect and analyze quantified data based on an automatically performed inspection, auscultation, percussion, and palpation. The proposed iApp system consists of an auscultation sensor, camera for inspection, and custom-built hardware for automatic percussion and palpation. Experiments were designed to categorize the eight abdominal divisions of healthy subjects based on the system multi-modal data. A deep multi-modal learning model, yielding a single prediction from multi-modal inputs, was designed for learning distinctive features in eight abdominal divisions. The model's performance was evaluated in terms of the classification accuracy, sensitivity, positive predictive value, and F-measure, using epoch-wise and subject-wise methods. The results demonstrate that the iApp system can successfully categorize abdominal divisions, with the test accuracy of 89.46%. Through an automatic examination of the iApp system, this proof-of-concept study demonstrates a sophisticated classification by extracting distinct features of different abdominal divisions where different organs are located. In the future, we intend to capture the distinct features between normal and abnormal tissues while securing patient data and demonstrate the feasibility of a fully telediagnostic system that can support abnormality diagnosis.

## 1. Introduction

Telemedicine-based diagnosis requires sufficient information; to this end, clinical decision support systems (CDSSs) that utilize visual information have been proposed (Sutton et al., 2020). Disease symptoms often contain features that are not routinely recognized by patients but can be identified through indirect inspection or diagnosis by medical professionals. However, additional medical diagnostic tools are needed for improving the CDSSs' performance (Belard et al., 2017; Zikos and DeLellis, 2018). Moreover, since the COVID-19 pandemic, telemedicine has garnered increasing attention (Bashshur et al., 2020; Ohannessian et al., 2020; Portnoy et al., 2020; Vidal-Alaball et al., 2020), and basic diagnostic tools (e.g., classical examination) have become the most important components of a comprehensive framework (Belard et al., 2017; Sutton et al., 2020; Fuchtmann et al., 2021). Medical researchers have been increasingly interested in developing objective systems that do not use blood samples (Jeong et al., 2018). Automatic and effective evaluation systems are desired that will be able to deliver basic inspection information as successfully and accurately as possible. When such evaluation systems are used in telemedicine and remote patient monitoring, it is desired to deliver the acquired information, such as hospital-level information (Wasylewicz and Scheepers-Hoeks, 2019). The most basic approach to medical diagnosis is physical examination (Verghese et al., 2011). A diagnostic process starts by detecting abnormal symptoms outside and inside the body: changes in the skin surface color and fissure, changes in tactile sensation, changes in sounds generated by the tissue itself or in response to external stimuli.

Inquiry, inspection, auscultation, percussion, and palpation are five traditional physical examination methods that have been used in the clinical field (Narula et al., 2018). In particular, inspection, auscultation, percussion, and palpation are the most basic non-invasive physical diagnostic methods that have been used since 1761. Abdominal physical examination is essential for clinical observations of signs and causes of diseases of the patient's abdomen; this examination includes inspection, auscultation, percussion, and palpation methods. This examination is performed for determining abnormalities by judging the size, shape, positional mobility, and consistency of abdominal organs (Ball et al., 2017; Jarvis, 2018). Abdominal inspection is the most basic test and is used for determining changes of the skin surface (Hayden et al., 2021). Diagnosis is made by observing the skin color, quality or contours, lines or scars, observations of the blood vessels' network, the shape of the belly button, and the abdomen's surface movement patterns. Systemic and organ-specific changes accompany some diseases; therefore, a differential diagnosis can be improved through abdominal shape or color changes (Floch, 2019). For example, in the case of liver cirrhosis, changes such as caput medusae, where enlarged veins are observed in the abdominal wall, can be observed. In addition, bruising of subcutaneous adipose tissue of the abdominal wall around the navel (Cullen sign) or side (Grey Turner sign) is an indication of pancreatitis, suggesting intraperitoneal bleeding (Wright, 2016). Auscultation is essential for physical examination and helps to diagnose various diseases. Abdominal auscultations examine intestinal sounds in the gastrointestinal system, for determining sound irregularities that are inconsistent with expected sound propagation, and for classifying these abnormal sounds caused by pathological changes in the body system into several specific types (Gade et al., 1998). In particular, a rebounding sound generated in response to an external stimulus may inform about the condition of the body's internal organs. Abdominal examination may indicate peritonitis or paralytic ileus if bowel sound decreases; however, diarrhea, gastrointestinal bleeding, or mechanical ileus may also be suspected if the bowel sound increases (Podolsky et al., 2015). The usefulness of auscultation is limited but helpful in diagnosing abdominal occlusion, which may be suspected if the metallic sound is auscultated (Podolsky et al., 2015). For example, clinicians showed an accuracy of 84.5% in ileus detection by listening to bowel sounds (Gu et al., 2010). Percussion is performed by administering a mechanical impact, using a percussion hammer or fingers. Medical personnel determine the position, size, consistency, and boundaries of fundamental organs and their associated pathologies, by interpreting the sound's amplitude and pitch (Ayodele et al., 2020). The difference between normal and abnormal tissues appears to be a phenomenon such as “clear and long-lasting sound described as resonance,” owing to impedance discrepancies in the inspected area (Yernault and Bohadana, 1995). Palpation allows to determine tissue abnormalities as differences in stiffness between normal tissues and surrounding tissues, by measuring the tissues' physical properties (Ahn et al., 2012; Yasmin and Sourin, 2012a,b, 2013).

These traditional physical examination methods have been used to date owing to the advantages of speed and convenience (Hsu et al., 2020). However, diagnosis by inspection, auscultation, percussion, and palpation depends on the subjective interpretation of the test results by medical personnel (Ferguson, 1990). Therefore, the diagnosis results vary across individual clinicians and are often discordant (Khani et al., 2011). Current inspection, auscultation, percussion, and palpation techniques are used as pre-examination tools by medical personnel and are generally not considered reliable diagnostic methods (Wu et al., 2010; Durup-Dickenson et al., 2013; Mota et al., 2013). Of course, there are abdomen follow-up tests that use ultrasound, X-rays, computed tomography (CT), and/or magnetic resonance imaging (MRI); however, these technologies remain inaccessible to a wide population owing to their limited accessibility and high cost. Various attempts have been made to overcome this limitation, such as quantifying the performance of specific and effective initial lesion screening tests using objectively collected basic clinical information, and/or using machine-learning techniques (Sajda, 2006; Wu et al., 2010; Hunt et al., 2019). In particular, many studies have provided strong evidence of the potential for future use of computational analysis in the diagnosis of abdominal diseases and disorders (Inderjeeth et al., 2018).

The relationship between effective regulation of the bowel and upper abdominal suppleness using a load cell and a magnetic position sensor was evaluated (Kato et al., 2012). The study found an elastic relationship between the reactive force and the pressed depth at the palpation points. Hsu et al. (2020) proposed an integrated system based on a force sensor and a camera, for quantifying the palpation pressure and location simultaneously, with the idea that this system will serve as a reference for digital abdominal palpation devices. Several research groups have studied automatic percussion systems based on surface exciters for generating percussive sound inputs, or push-pull solenoid actuators for generating mechanical impulses (Rao et al., 2018; Ayodele et al., 2020). The automatic percussion instrument based on the solenoid actuator achieved a test accuracy of 94% for the classification of the three thoracic sites to elicit dull, resonant, and tympanic signals (Ayodele et al., 2020). Krumpholz et al. (2021) proposed a robotic end-effector for mechanical percussion to classify lung and non-lung samples. They proved the feasibility of telemedical percussion with a classification accuracy of 71.4%. However, the above studies focused either on quantitative tests and the development of guidance systems or on the automation of single tests. In addition, the lack of subject-wise tests made it difficult to evaluate the generalization performance of the systems. It appears that there are limitations precluding the development of a comprehensive physical examination and diagnosis system.

In other fields, attempts have been made to determine the target object's properties based on the physical hitting characteristics (Gong et al., 2019; Ryu and Kim, 2020b,c). An automatic classification system has been proposed that identifies the characteristics of a surface based on the inertial and audio signals measured when tapping the surface (Ryu and Kim, 2020a). However, further considerations should be addressed before this method can be applied in medical analysis; these considerations necessitate careful experimental design and a data analysis framework. Research on artificial intelligence (AI)-based automated decision-making physical examination systems has not yet been conducted. Hence, to automate abdominal physical examinations and perform remote promotions, further research is needed.

Improved inspection, auscultation, percussion, and palpation methods that incorporate the latest technologies for automating abdominal physical examination and remote promotion can be achieved by improving the sensitivity, specificity, and reproducibility of these diagnostic methods. To this end, the following sequential build-up process is required: (1) development of automated inspection, auscultation, percussion, and palpation device; (2) validation of device performance and data; and (3) large-scale clinical trials. As a proof-of-concept study prior to a large-scale clinical trial, this study focuses on determining the feasibility of the proposed approach. This is achieved by developing a system for automating and quantifying inspection, auscultation, percussion, and palpation, extracting distinct features from test data, and investigating the possibility of classifying specific locations on the abdomen. The evaluation is performed by system configuration, data acquisition, and analysis through deep multi-modal learning. Custom-built percussion and palpation devices were used to quantify the stimuli applied to each subject during the evaluation process. Taking a nine-division abdominal examination as an example, we attempted to classify the anatomical landmarks of the abdomen into eight divisions, excluding the umbilical region. The nine-division scheme was adopted because a more detailed diagnosis is accessible when the abdomen is divided into nine divisions rather than four quadrants (Floch, 2019). The measurement in the umbilical region was excluded in the current study because the geometry around the navel was different from that of the other eight divisions. Pilot test results, obtained from 30 healthy young subjects, were evaluated for classification accuracy, sensitivity, positive predictive value (PPV), and F-measure, using epoch-wise and subject-wise methods. Through an automatic examination of the iApp system, this proof-of-concept study demonstrates a sophisticated classification by extracting distinct features of the abdominal divisions where different organs are located. In the future, we intend to capture the distinct features between normal and abnormal tissues while securing patient data and demonstrate the feasibility of a fully telediagnostic system that can support abnormality diagnosis.

## 2. Materials and Methods

### 2.1. Participants

Thirty adults (15 females; age, 19–26 years) participated in this study. None of the participants had disabilities based on abdominal percussion and palpation and did not report pain in response to weak skin stimulation. Table 1 summarizes the information on the subjects who participated in the study. They were not restricted to any conditions such as ingestion and bladder. The Hallym University Institutional Review Board approved this study, and all participants provided written informed consent (HIRB-2021-057-1-R).

**Table 1:** Information on the subjects who participated in this study: BMI, body mass index; SD, standard deviation.

		**Age, y**	**Height, cm**	**Weight, kg**	**BMI**
Female (n=15)	Mean	22.1	162.0	57.5	21.9
	SD	1.6	5.4	10.2	3.4
Male (n=15)	Mean	21.7	175.0	71.5	23.3
	SD	2.0	5.5	11.1	3.2
All (n=30)	Mean	21.9	168.5	64.5	22.6
	SD	1.8	8.5	12.6	3.3

### 2.2. Experimental Design

**Figure 1:**
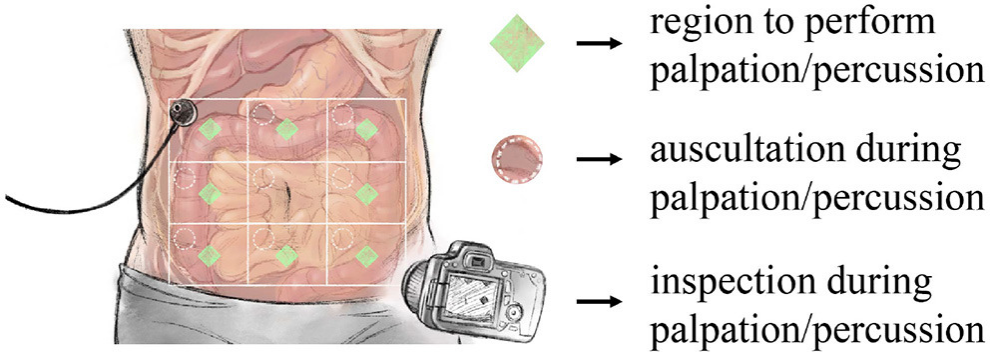
Overview of the designed experiment. iApp-based signal collection was performed for eight areas (excluding the navel-containing area).

The experimental process started with abdominal percussion and ended with abdominal palpation. As shown in Figure 1, for each subject the abdomen was divided into nine square areas, and iApp collected reaction signals (in response to stimulation) from all areas except the navel-containing one.

**Figure 2:**
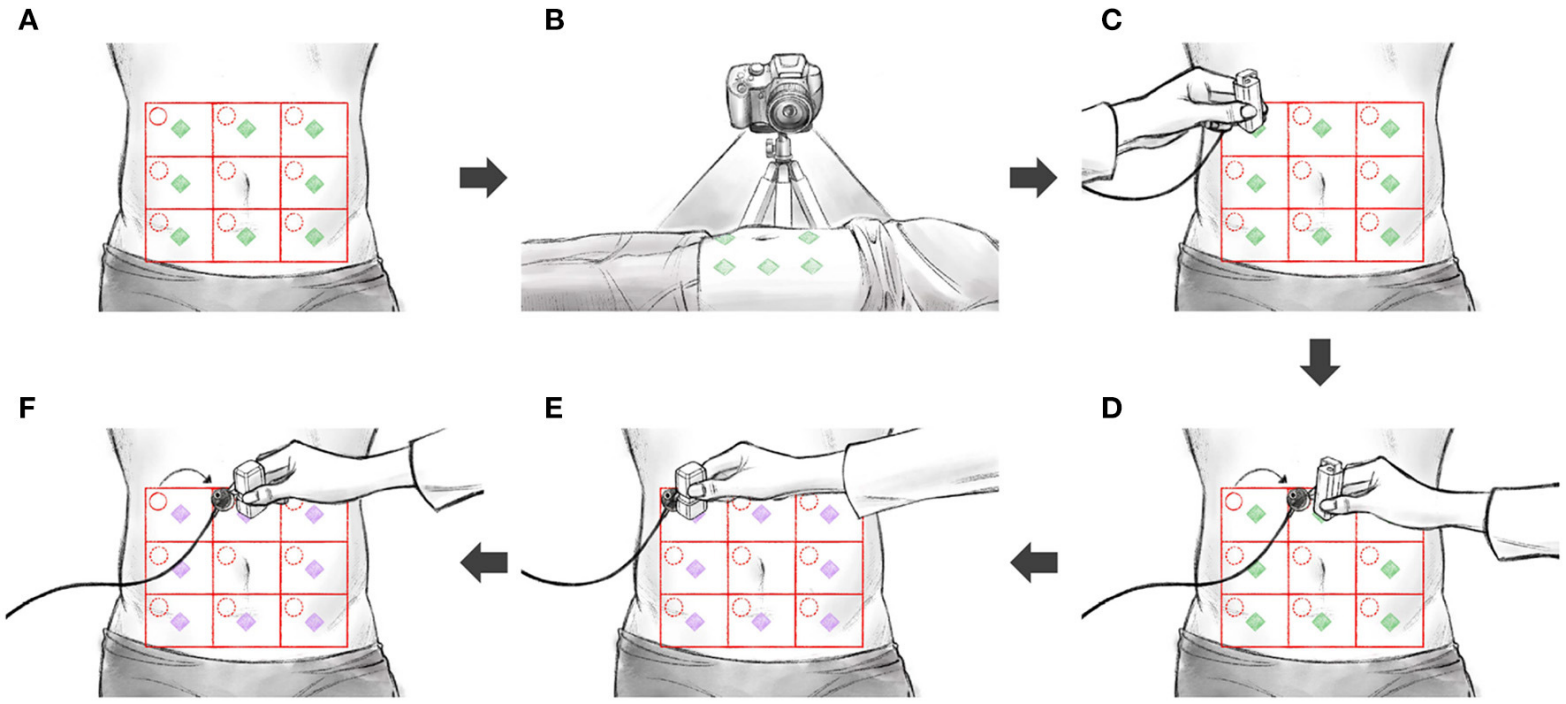
The sequence of experiments: **(A)** divide the abdomen into nine divisions, **(B)** set the camera, **(C)** install the auscultation sensor and perform percussion, **(D)** repeat percussion, **(E)** install the auscultation sensor and perform palpation, **(F)** repeat palpation.

Figure 2 shows the full experimental data collection process, which consisted of the following steps:

(a) Divide the abdomen into nine equal square-like areas. The participant was in the supine position, with the abdomen exposed. The abdomen was divided into a 3× 3 grid, centered on the belly button. The middle compartment (navel-containing) was not used in our measurements.
(b) Set the camera. A camera was positioned so as to take videos of all nine divisions.
(c) Install the auscultation sensor and perform percussion. An auscultation sensor (physiological sound transducer) was attached to the diagonal right corner of the subject's abdomen, at the target position. After the attachment, percussion was performed at the measurement position, using a percussion device. The operator placed the percussion device in the measurement position and prevented the subject from moving during the measurement; each measurement took 15 s. After the initial 3-s-long stabilization, the percussion device first hit the subject's abdomen at the measurement position. After 5 s, the percussion device consecutively hit the same position in 0.5-s-long intervals.
(d) Repeat percussion. Eight abdomen divisions were measured for each consecutive replicates. The sequence of measurements from division 1 to division 9 constituted one cycle; overall, five measurement cycles were completed. When moving over to another division, the physiological sound transducer moved as well. The transducer's attachment sites were the same for each division.
(e) Install the auscultation sensor and perform palpation. A physiological sound transducer was attached to the diagonal right corner of the subject's abdomen, at the target position. After the attachment, palpation was performed at the measurement position, using a palpation device. The operator placed the palpation device in the measurement position and prevented the subject from moving during the measurement; each measurement took 15 s. After the initial 3-s-long stabilization, the subject's measurement position was pressed for 1.5 s and released for 1.5 s. After 5 s, the measurement position was pressed again for 1.5 s, and the palpation device was immediately detached from the patient's body by the operator.
(f) Repeat palpation. Eight abdomen divisions were measured, each in six consecutive replicates. The sequence of measurement from division 1 to division 9 constituted one cycle; overall, five measurement cycles were completed. When moving over to another division, the physiological sound transducer moved as well. The transducer's attachment sites were the same for each division.

### 2.3. Experimental Setup

#### 2.3.1. System

**Figure 3:**
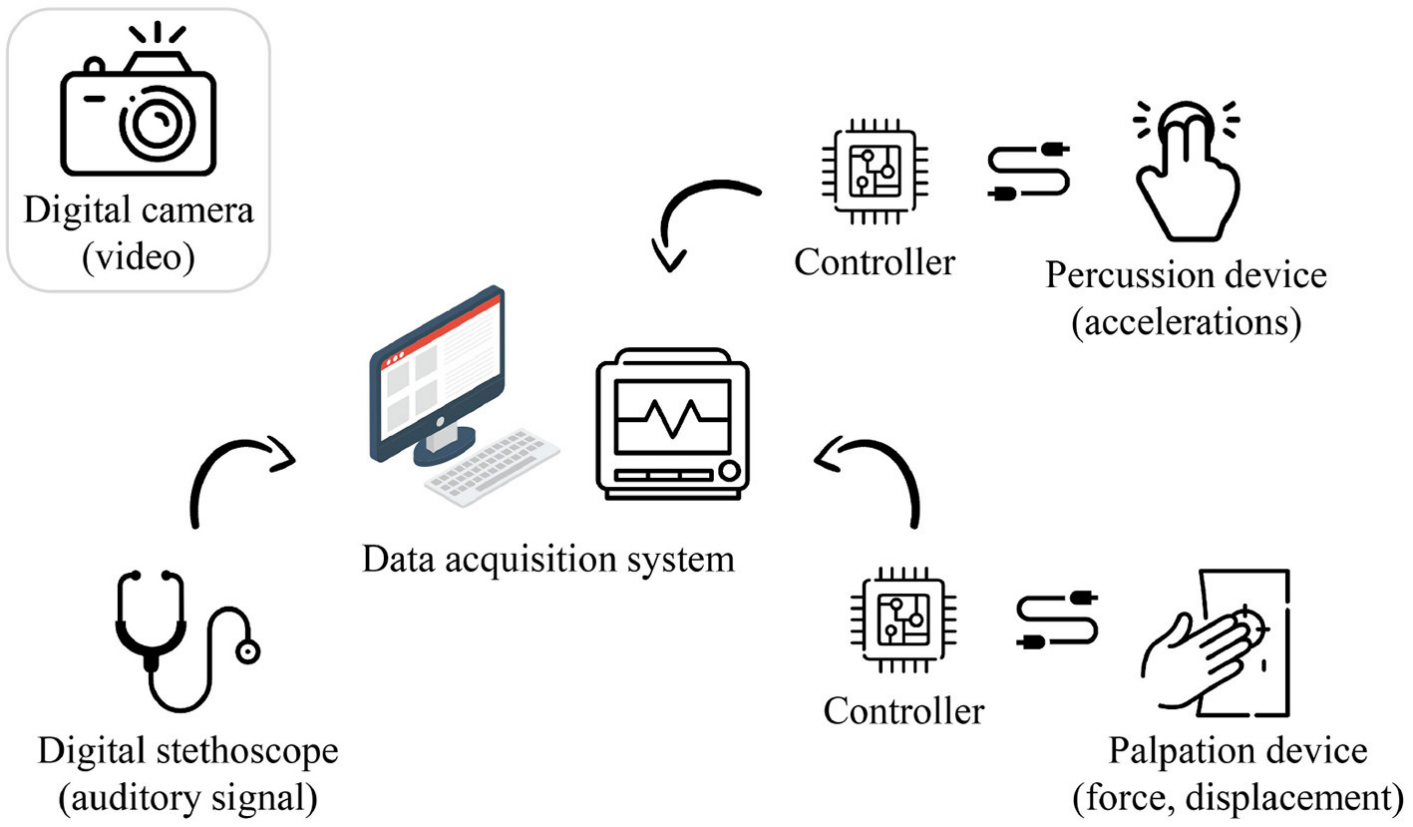
Hardware configuration of the proposed system (image: Flaticon.com).

The measurement system was configured for the data collection, as shown in Figure 3. A commercial camera (EOS M6 Mark II, Canon) and a commercial physiological sound transducer (TSD108, BIOPAC Systems) were used for inspection and auscultation, respectively. Custom-built hardware was developed for palpation and percussion.

The palpation device consisted of a potentiometer-equipped linear actuator (PQ12, Actuonix), a force sensor (CS8-10N, SingleTact), and three-dimensional (3D) printed housing for incorporating these components. The linear displacement and reactive force of the actuator were measured while the linear actuator in contact with the subject's abdomen was pushed and released. The stroke of the linear actuator was 20 mm, and the full-scale input of the force sensor was 10 N. It was possible to measure a reaction force of up to approximately 10 N while pressing the abdomen to a depth of approximately 20 mm. The dimensions of the fabricated palpation device were approximately 25 × 40 × 50 mm (length, width, and height), including the housing.

The percussion device consisted of a solenoid actuator (JB-0826B, Yueqing Gangbei Electric), a 3-axis accelerometer (ADXL343, Analog Devices), and 3D printed housing for incorporating these components, which utilized the mechanisms developed in our previous study (Ryu and Kim, 2020c). The solenoid actuator was loaded and then unloaded to apply a physical impact to the abdomen (approximately 10 N), and the resulting accelerations (-6 to 6 G) were measured. The dimensions of the manufactured percussion device were approximately 30 × 3 × 50 mm (length, width, and height), including the housing.

The auscultation, percussion, and palpation processes were controlled using a data-acquisition system (MP150, BIOPAC). The percussion and palpation examinations were controlled by MP150, sending an experimental start signal to an electronic circuit (including a microcontroller unit and a motor drive), constructed for controlling the actuators. The auscultation, percussion, and palpation data were collected through the analog channels of the MP150 system. The auscultation, percussion, and palpation data were sampled at 10,000, 312, and 312 Hz, respectively. Meanwhile, an inspection was performed by capturing a video of the entire abdomen using the camera, and these imaging data (in the form of a video clip) were stored in a micro secure digital (SD) memory.

#### 2.3.2. Data Collection

**Figure 4:**
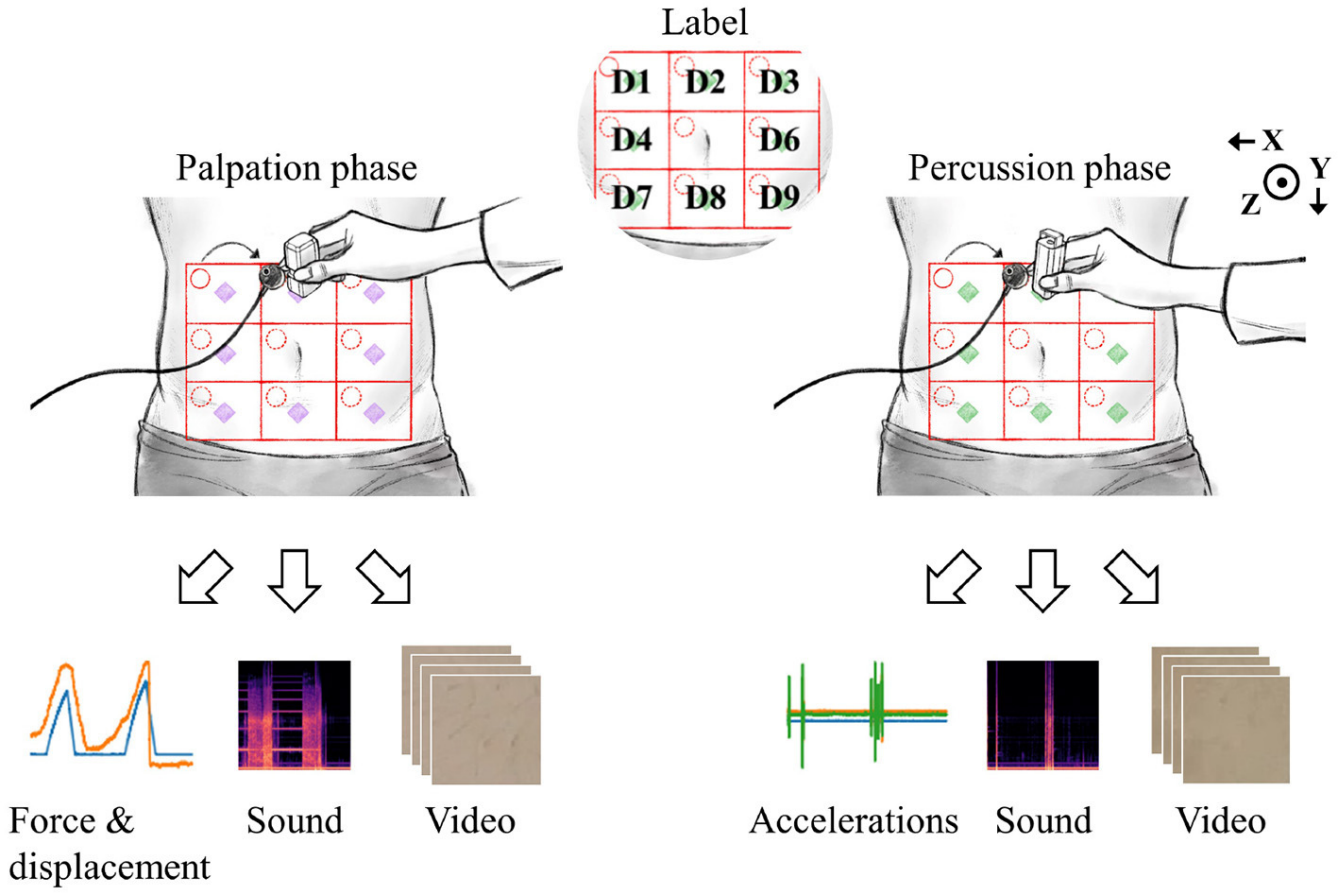
Data were collected by palpation and percussion. Each examination yielded three types of signals.

Figure 4 shows an example dataset collected in the experiments. The nine abdomen divisions were labeled D1 to D9, excluding D5 where the navel was located. The displacement and reaction force from the palpation device, sound from the physiological sound transducer, and video data captured by the camera, were recorded during palpation for each of the eight divisions. As described in the Experimental Design section, six consecutive replicate palpation tests were performed for each division, and the sequence of measurements was repeated five times. In other words, the dataset for the palpation test consisted of 7,200 samples (6 replicates × 8 divisions × 5 sequences × 30 subjects).

During the percussion test, 3-axis accelerations from the percussion device, sound from the physiological sound transducer, and video data from the camera were recorded. The target amount of data collected in the percussion test was the same as that obtained in the palpation test. However, 6,480 valid data samples per each test (percussion/palpation) were finally obtained because of the early termination of the experiment for several subjects. There were no cases in which the experiment was stopped because the subject complained of pain or voluntarily requested termination for any reason. However, a few of the experiments were terminated early by the intervention of the designer because of human error, hardware malfunction (power, circuit, performance problem, loss of wire connection, etc.), or errors in storing measurement results (data loss). The raw signals collected for each modality were as follows: inspection, 11.5-s-long video (1080p); auscultation, 11.5-s-long univariate time-series signal (115000, 1); percussion, displacement and force signals (3594, 2); palpation, x/y/z accelerations (3594, 3).

### 2.4. Data Analysis Framework

#### 2.4.1. Learning Model

**Figure 5:**
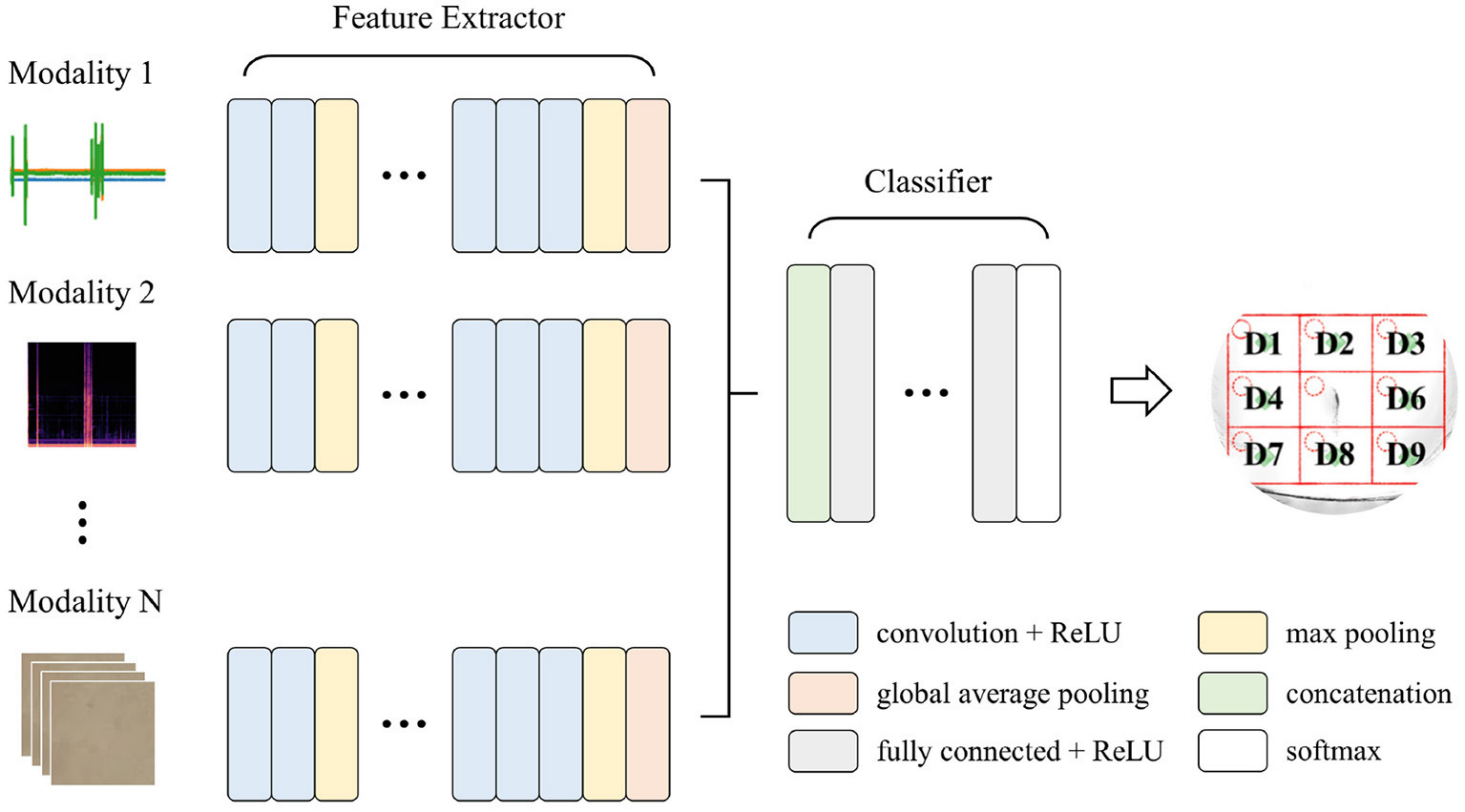
The proposed deep multi-modal learning architecture. The network has a split architecture with separate branches for each modality, which are then concatenated.

Convolutional neural networks (CNNs) have been widely used for time series, imaging, and video data analysis in various research areas, owing to their capability to learn both local and global features with relatively low computational cost, compared with recurrent neural networks (LeCun et al., 1995; Ronao and Cho, 2016). This study adopted a multi-input deep learning architecture based on CNNs, which yielded a single prediction from heterogeneous input signals. The goal was to learn distinctive features from the iApp-collected information, for predicting the eight abdomen divisions. Figure 5 schematically shows the proposed architecture for deep multi-modal learning, which incorporates features extracted from different modalities. The architecture comprises two main parts: a feature extractor and a classifier. The feature extractor was designed to extract features from each input signal stream (inspection, auscultation, percussion, and palpation) independently (Simonyan and Zisserman, 2014).

For each path, appropriate layers among one-dimensional (1D) convolutional, two-dimensional (2D) convolutional, and 3D convolutional layers were applied according to the shape of the input signal. Each path consisted of ten convolutional layers and four max-pooling layers, with a pooling size of 2. For the convolutional layers in all paths, the number of filters was 16, 16, 32, 32, 64, 64, 64, 128, 128, and 128, and the kernel size and stride were 3 and 1, respectively. A global average pooling layer was applied at the end of each path so that the output of the feature extractor was 128 per modality. All features from different input modalities were then concatenated, followed by three fully connected (dense) layers with 512, 128, and 8 nodes. A rectified linear unit (ReLU) was used as an activation function, except for the output node that used softmax activation. Note that we tried to simplify the model's complexity as much as possible (considering the future embedded implementation), while at the same time ensuring a good performance.

In addition, we applied the following regularization techniques to improve the model's generalization performance: (1) kernel regularization was applied to all convolutional and dense layers; (2) a batch normalization layer was added immediately after each convolutional layer; and (3) a dropout of 0.5 was applied to the first two dense layers.

#### 2.4.2. Pre-processing

For auscultation, the captured raw data were a univariate time-series signal. Short-time Fourier transform, which is important for characterizing abnormal phenomena of time fluctuation signals such as bowel sounds, was applied (Allwood et al., 2018). First, all data samples were normalized such that the signal was between -1 and 1. Second, a mel spectrogram image was generated by calculating the mel spectra for several windowed signal segments. The window and hop sample sizes were 2048 and 512, respectively. Finally, the generated image was resized to 64 × 64, and each pixel of the image was then divided by 255 for normalization. The resulting image, with the dimensions of 64 × 64 × 3, was used as the input representation for the 2D CNN path. For inspection, the captured raw data were a series of images (video). From the video data, we extracted two frames: immediately before the examination and immediately after the examination. Eight divisions were cropped individually from each image and then resized to 64 × 64. The two images were then stacked, and the shape of (2, 64, 64, 3) was formed as input representations of the 3D CNN path. The captured raw data were a multi-variate time-series signal for palpation, including the displacement of the linear actuator and the force measured during the palpation. All of the data samples, with the (3594, 2) shape, were standardized before usage as input representations of the 1D CNN path. Similarly, the raw data captured in percussion tests (three-axis acceleration signals, shape of (3594, 3) were standardized before being shown to the 1D CNN path.

#### 2.4.3. Data Partitioning and Evaluation Criterion

**Figure 6:**
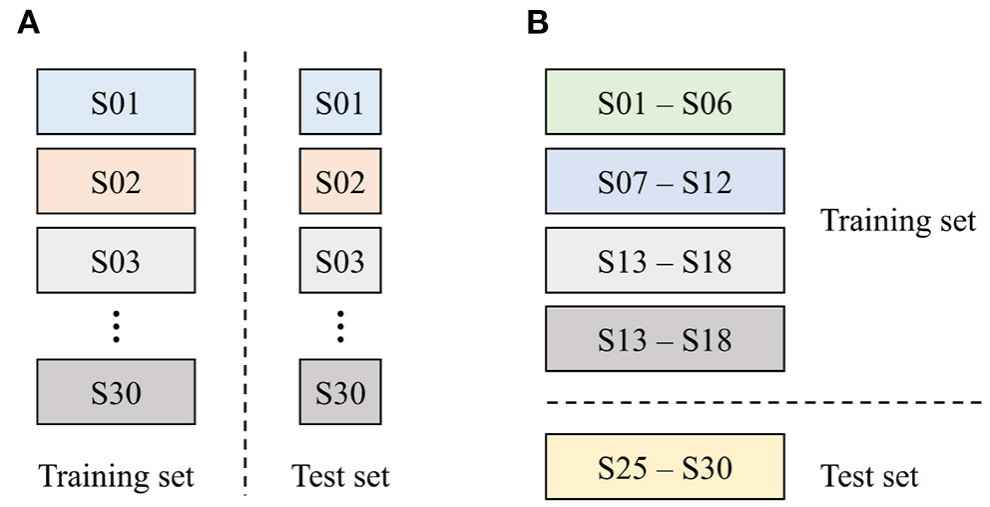
Data partitioning strategy used in this study. **(A)** Epoch-wise method and **(B)** subject-wise method.

Generally speaking, two types of data-partitioning methods are used for clinical data analysis: (1) subject-wise and (2) epoch-wise (or record-wise) partitioning. These are also called independent and non-independent methods, respectively (Supratak et al., 2017; Jiang et al., 2019). This study used the epoch-wise method for determining an appropriate model by investigating the effect of input modalities, while the subject-wise method was used for evaluating the generalization performance of the proposed approach. In the epoch-wise analysis, the entire dataset was separated into two independent sets: 70% of samples were used for training (4,520 cases), while the remaining 30% were used for testing (1,960 cases), as shown in Figure 6A. The data were used for evaluating the following three machine-learning models: (1) the palpation model used inspection, auscultation, and palpation data obtained in palpation tests as inputs (three input modalities); (2) the percussion model used inspection, auscultation, and percussion data obtained in percussion tests as inputs (three input modalities); and (3) the combined model consisted of the above two models, with six input modalities used as inputs.

In the subject-wise analysis, the entire dataset was divided into five groups (or folds), each containing six subjects, for 5-fold cross-validation, as shown in Figure 6B. In other words, a dataset of 24 subjects was used for training the model, while the data for the other six subjects were used for testing the model's performance. This process was repeated for each fold, yielding a total of five models. Because the models were evaluated using the subjects' data that have not been used for training (i.e., previously unseen data), the generalization performance of the proposed system could be assessed.

## 3. Results

### 3.1. Effect of the Input Modality

**Figure 7:**
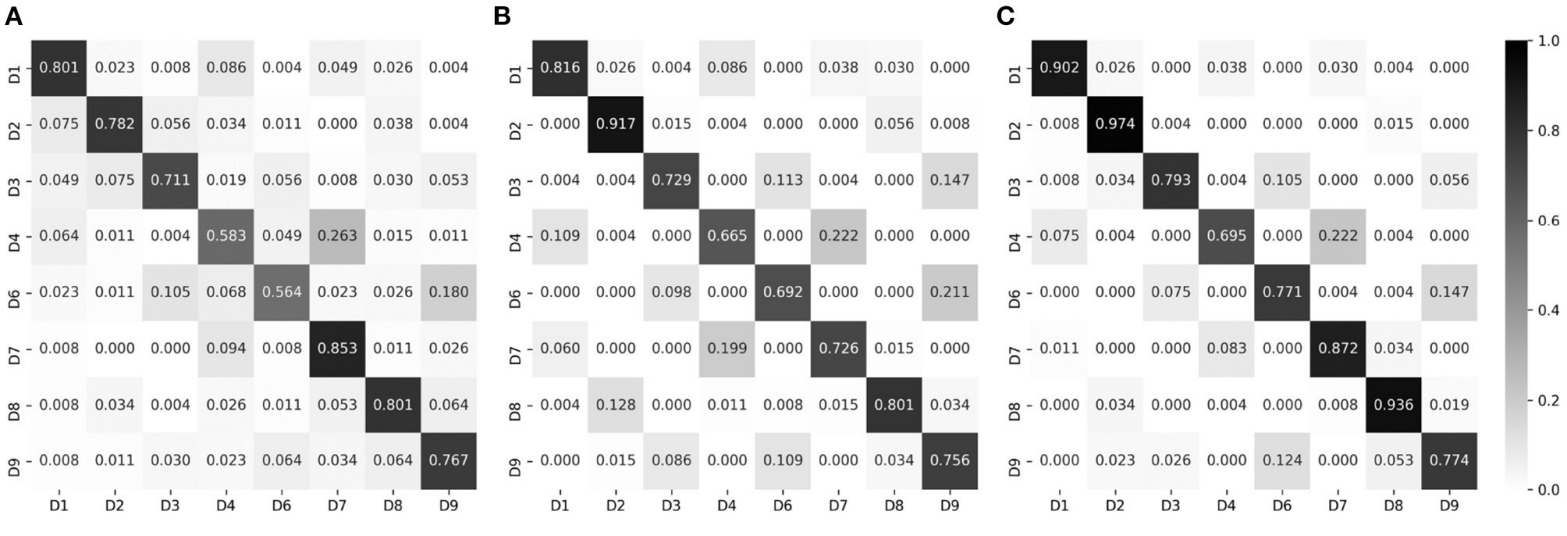
Normalized confusion matrices, for epoch-wise tests. Rows and columns indicate the actual and predicted classes, respectively. **(A)** Palpation only, accuracy of 73.26%. **(B)** Percussion only, accuracy of 76.27%. **(C)** Combined, accuracy of 83.98%.

Below, we report the input modality-based classification performance, in terms of the test accuracy. Figure 7A shows the test accuracy and the normalized confusion matrix for the palpation model (three input modalities). The overall test accuracy was 73.26%, and it was confirmed that correct divisions were in general found. The percussion model (three input modalities) achieved the test accuracy of 78.27%, as shown in Figure 7B. The combined model, which used all data (six input modalities) obtained through percussion and palpation, achieved the best test accuracy of 83.98%, showing a further performance improvement by complementing some difficult classification classes in the palpation and percussion-only models. Therefore, the proposed approach is feasible and it is desirable to employ a model that utilizes all of the information, such as inspection, auscultation, percussion, and palpation.

**Figure 8:**
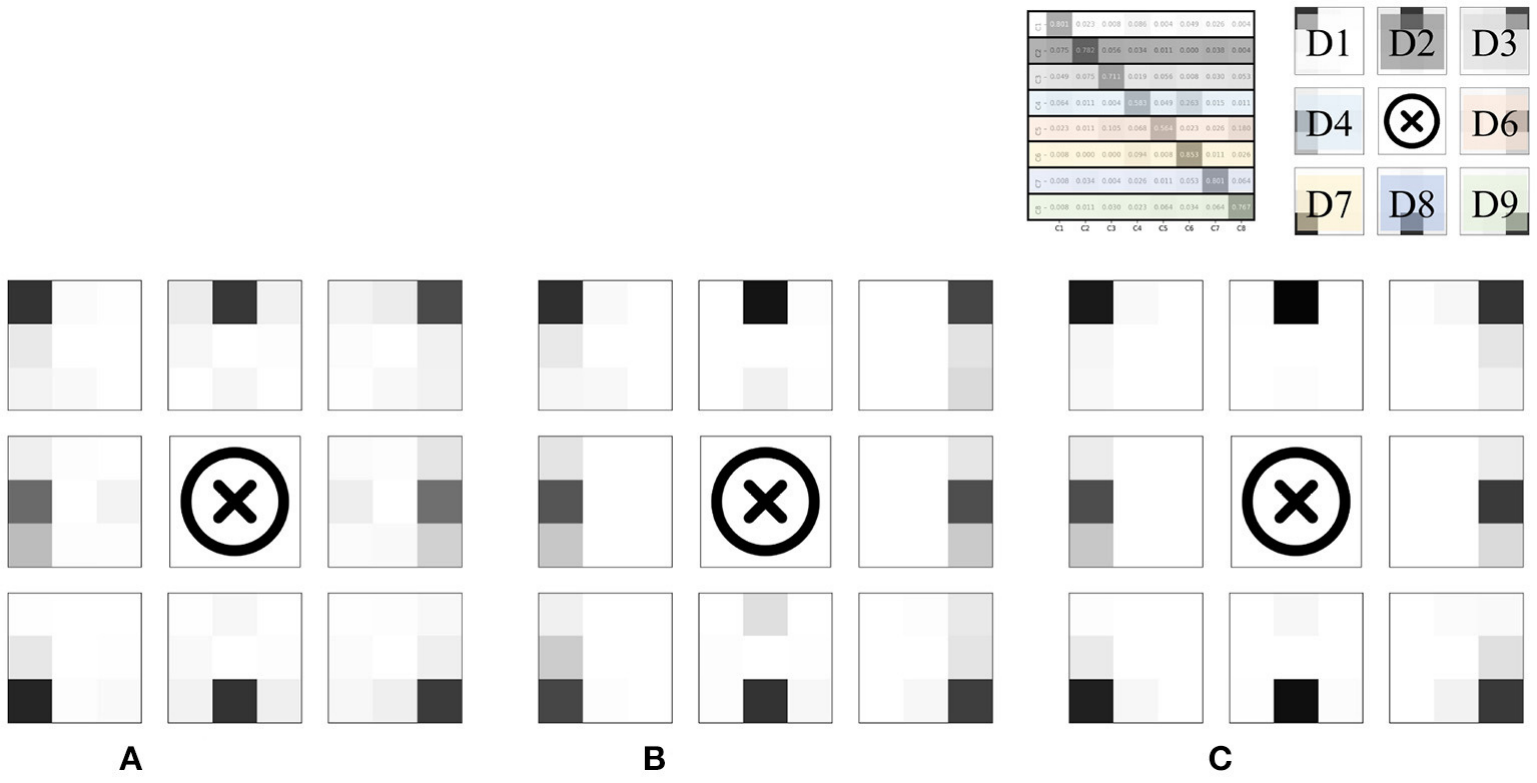
Heat map visualization of the confusion matrices in Figure 7. **(A)** Palpation only. **(B)** Percussion only. **(C)** Combined.

Meanwhile, non-negligible misclassification was observed even for the combined model. To intuitively determine which classes were misclassified, we converted each row of the confusion matrix into a 3 × 3 heat map and visualized it; the results are shown in Figure 8. Clearly, most misclassifications occurred for adjacent divisions. This phenomenon is discussed in the next section.

### 3.2. Generalization Performance

This section describes the results of subject-wise testing using a model that combines the percussion model and the palpation model. Table 2 summarizes the results of subject-wise 5-fold cross-validation in terms of PPV, sensitivity and F-measure. All of the metrics are weighted averages. In addition, the dataset collected in this study was completely balanced; therefore, the sensitivity was the same as the accuracy.

**Table 2:** Results of subject-wise testing.

	**Test set**					
	**Fold #1**	**Fold #2**	**Fold #3**	**Fold #4**	**Fold #5**	**Mean ±SD**
PPV	0.6501	0.6988	0.6717	0.6676	0.6782	0.6733 ± 0.0177
Sensitivity	0.6528	0.6882	0.6451	0.6535	0.6558	0.6591 ± 0.0168
F-measure	0.6469	0.6785	0.6432	0.6517	0.6475	0.6536 ± 0.0143
PPV^*^	0.8991	0.9124	0.9157	0.8980	0.8703	0.8991 ± 0.0179
Sensitivity^*^	0.8931	0.9069	0.9128	0.8897	0.8621	0.8929 ± 0.0197
F-measure^*^	0.8917	0.9070	0.9133	0.8881	0.8634	0.8927 ± 0.0194
PPV^**^	0.8794	0.8791	0.8825	0.8677	0.8442	0.8706 ± 0.0158
Sensitivity^**^	0.8743	0.8715	0.8688	0.8611	0.8333	0.8618 ± 0.0167
F-measure^**^	0.8712	0.8715	0.8716	0.8592	0.8334	0.8614 ± 0.0165

*The metrics with single and double asterisks denote measurements based on the three-adjacent accuracy and vertical nearest accuracy schemes, respectively. SD, standard deviation; PPV, positive predictive value*.

For the first three rows (results based on top-1 accuracy), PPV, sensitivity, and F-measure did not deviate significantly across the different folds. However, the average accuracy (here, sensitivity) was 65.91%, slightly lower than the result for epoch-wise testing. Based on these results alone, it needed more investigation to determine whether the model's performance is sufficiently generalizable.

We defined two new terms for further analysis of the results: *three-adjacent accuracy* and *vertical nearest accuracy*, inspired by the top-N accuracy. *Three-adjacent accuracy* was defined as the frequency with which the predicted class was included in the correct answer class and the two divisions closest to the correct answer class (both horizontal and vertical). For example, if the prediction was D1, D2, or D4 when the actual answer should have been D1, it was treated as the correct answer. To consider another example, if the prediction was one of D7, D8, or D9 when the actual answer should have been D8, it was treated as the correct answer.

The *vertical nearest accuracy* was defined as the frequency with which the predicted class was correct and in the closest vertical division. For example, if the prediction was either D1 or D4 when the actual answer should have been D1, it was treated as the correct answer. To consider another example, if the prediction was only D8 when the actual answer should have been D8, it was treated as the correct answer. D5 was removed because of the belly button location.

**Figure 9:**
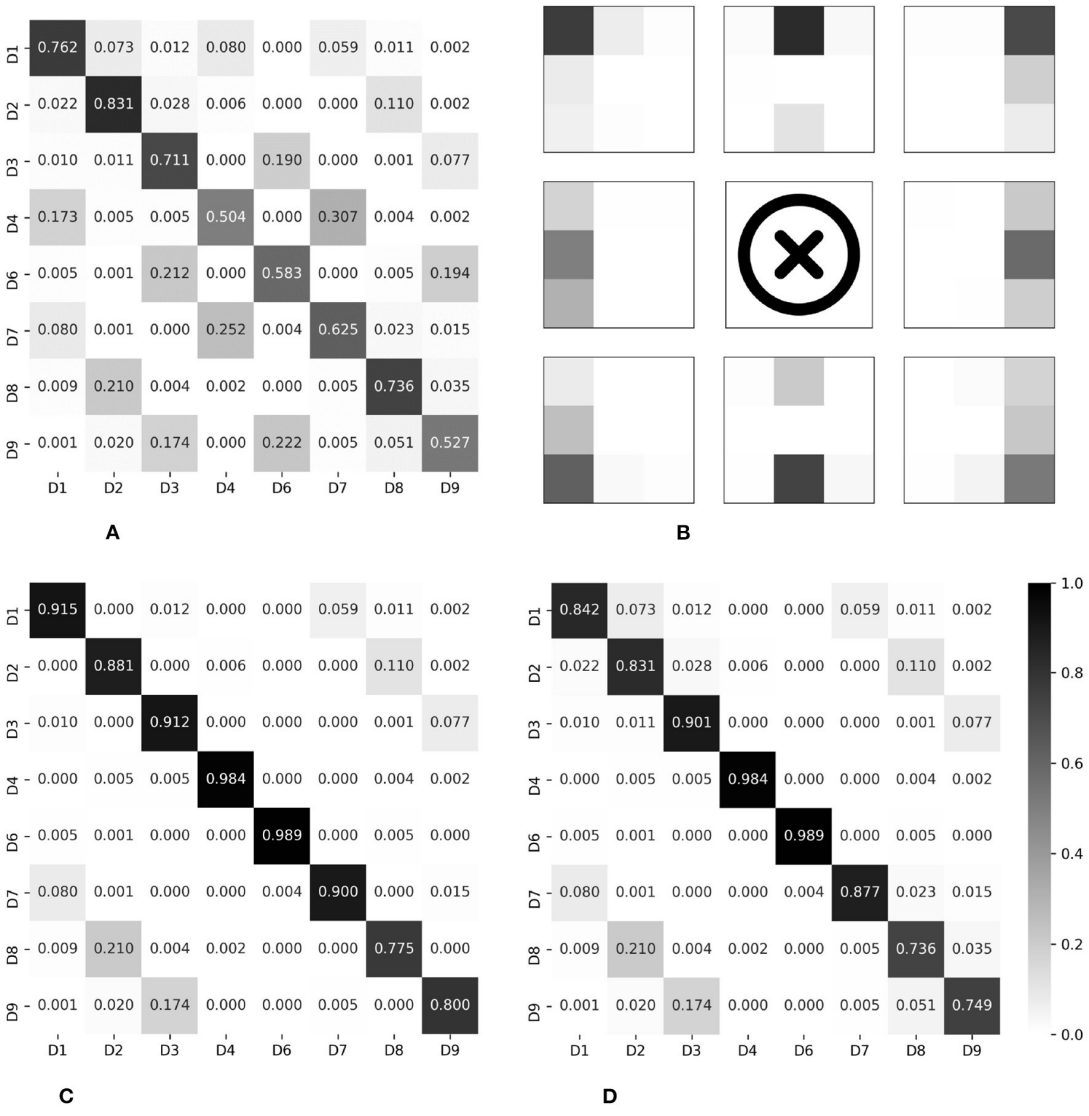
Normalized confusion matrices and heat map visualization, for subject-wise testing. Rows and columns indicate the actual and predicted classes, respectively. **(A)** Top-1 accuracy, 65.79%. **(B)** Visualization of Top-1. **(C)** Three-adjacent accuracy, 89.46%. **(D)** Vertical nearest accuracy, 86.36%.

The results evaluated using the two newly defined terms are summarized in the place marked with an asterisk in Table 2. Compared to when viewed from the perspective of top-1 accuracy, it was confirmed that the performance improved dramatically. In other words, most of the misclassifications occurred between adjacent divisions, consistent with the results of epoch-wise testing. Figure 9 shows the confusion matrices and heat map visualization of the above results. In particular, Figures 9B–D confirm that most of the predictions occur near the correct division, verifying that good performance can also be achieved in subject-wise testing. In summary, the proposed system can be applied to new subjects who were not a part of the training dataset. More details on the misclassification can be found in the Discussion section.

## 4. Discussion

### 4.1. Misclassifications

In this study, epoch-wise analysis was performed for determining the structure of a machine-learning model suitable for regional classification using inspection, auscultation, percussion, and palpation data. As a result, a deep multi-modal learning architecture that utilizes the entire data generated through percussion and palpation tests (the combined model) was proposed, and the model's effectiveness was confirmed. Subject-wise analysis was performed based on the combined model for validation, and the results of the analysis demonstrated that the developed methodology can be used as a pre-screening tool for determining the abdomen divisions.

As a result of the experiment, in particular, two main points were found in the pattern of misclassification. First, misclassification in the vertical direction occurred for the D1/D4/D7, D3/D6/D9, and D2/D8 groups. Anatomically, on the abdomen's right side, D1/D4/D7 is where the ascending colon is located and the ascending colon passes from D7 to D1 among the divisions presented in this paper. The confusion matrix for the top-1 accuracy shows a series of misclassifications between D1/D4/D7, as shown in Figures 9A,B. Had the area where the ascending colon is located been measured directly or indirectly, the series of these three divisions would have reflected the characteristics of the ascending colon. This phenomenon was also observed for the D3/D6/D9 series. This area is anatomically a partitioned surface in which the descending colon is located. Therefore, for the D3/D6/D9 series, misclassification was observed, similar to the series of the ascending colon. Here, it is difficult for the D2/D8 series, excluding the navel area, to represent anatomically the same organ. There are individual differences, but D2 includes the stomach or the transverse colon, and D8 includes the small intestine. From the algorithm view, the D2/D8 series may have similar stiffness values and/or similar resonance sounds. The D2/D8 series should be discussed with the addition of objective long-term verification, with more data and images.

Second, some misclassification phenomena occurred for adjacent divisions. Participants had physical characteristics of 168.5 ± 8.5 cm and 64.5 ± 12.6 kg (mean/SD). Therefore, anatomically, the distinction centered on the belly button will differ in the microscopic organ positions of individual subjects. The developed algorithm would have recognized this acceptable difference as information, and this would have resulted in uncertain divisions. We predict that this difference caused misclassification near the right and left divisions. On the other hand, misclassification that occurs in the outer area, except for vertical and horizontal neighbors, is an error that does not reflect the characteristics during the learning process. The generalization performance can be supplemented by learning more and various age groups, which requires additional experiments.

### 4.2. Limitations and Future Conceptual Work

**Figure 10:**
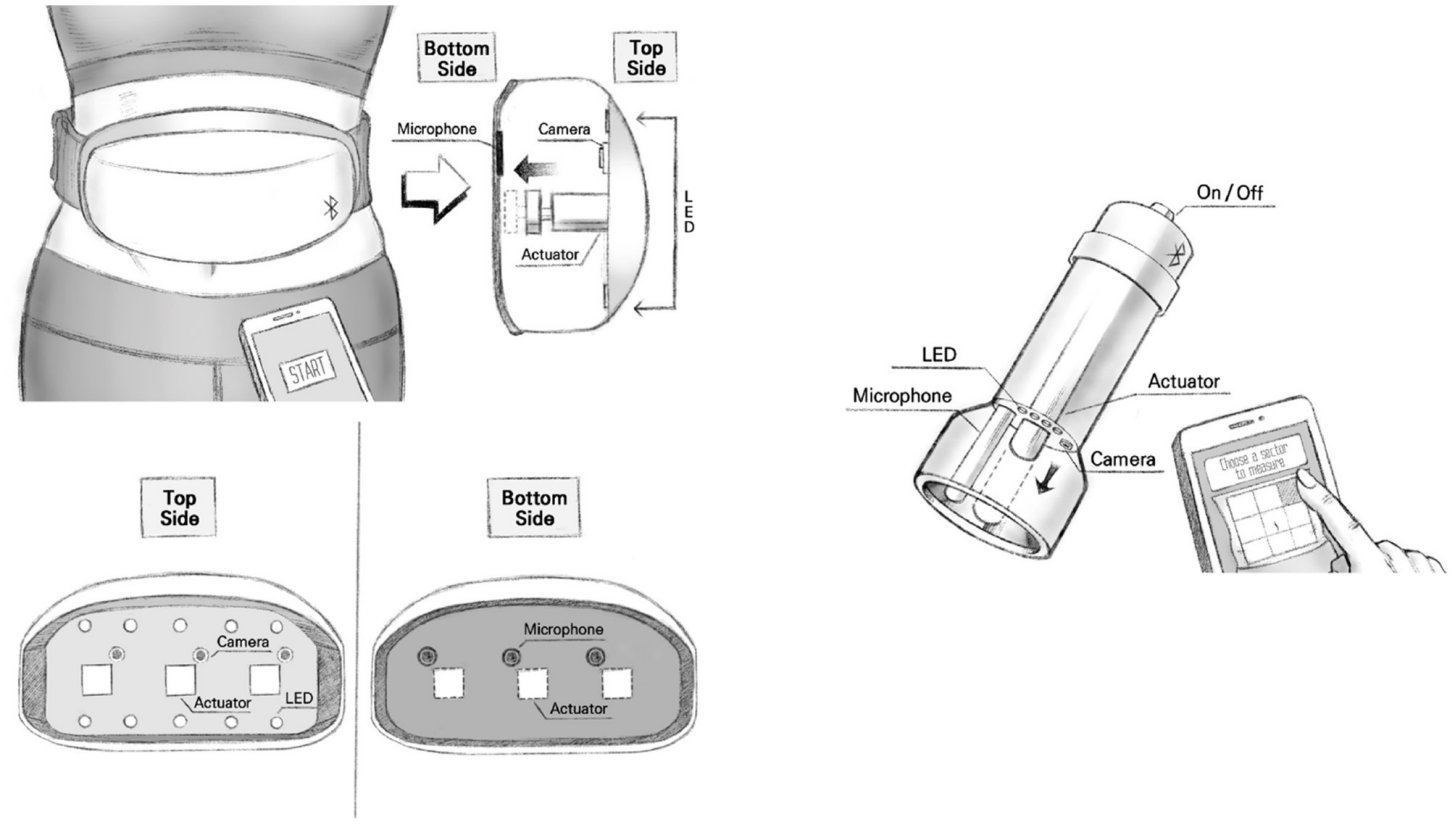
The proposed system concepts for wearable and portable devices.

Autonomous classification frameworks that allow remote progression of classical examination modalities have scarcely been studied. In this study, the investigation of the pre-development of the iApp system in the form of a wearable device was primarily focused on verifying whether the proposed approach can be used for robust and reliable assistance. For this system to develop into a wearable form, an auscultation sensor, a percussion sensor, a palpation sensor, and a camera must be integrated within a single device. The dimensions of individual devices must be sufficiently small to be worn and/or be portable and must be operated in low-cost embedded environments. Compact-size integrated hardware systems can be made sufficiently configurable by utilizing very small or micro- actuators/sensors (as in this study). Potential development of similar wearable or portable devices is proposed, as shown in Figure 10.

Ideally, the model should identify eight abdomens regardless of the subject's BMI, age, gender, weight, height, ingestion, and bladder condition. In general, when patients first visit the hospital, their physiological conditions, such as ingestion and bladder, may differ. For instance, the patient may be in a fasting or postprandial state. Therefore, doctors are trained to establish criteria for abnormalities, even in various patient physiological conditions. In this study, rather than limiting the various patient environments for measurement standardization, we attempted to match the initial treatment stage in which patients and clinicians come into contact. However, the subjects evaluated in the current study were young with an average BMI of 22.6, which falls in the normal range. Thus, experiments on more diverse subject groups are required to improve the analysis's generality further. In addition, we performed percussion and palpation only at the center of each division, excluding the umbilical region. To obtain more accurate results, it is desirable to perform examinations at various locations within each division, including the umbilical region. Based on these considerations, new analyses and additional experiments need to be conducted in the future.

In a recent study, the authors confirmed that the deep multi-modal learning model also works well for embedded machines (Ryu and Kim, 2020a). Considering that the complexity of the neural network proposed in this paper is relatively light, it is expected that real-time predictions will be possible even using embedded environments. Meanwhile, to use the proposed model in an embedded environment, the computational cost should be sufficiently low to be acceptable for embedded devices. These issues will be addressed in more depth in future studies.

In this trial, palpation was limited to approximately 2 cm, corresponding to light palpation. Typically, palpation can be divided into light and deep palpations. Generally, light palpation is performed first, followed by deep palpation. Light palpation is useful for detecting abnormalities in the abdominal surface, and texture, tenderness, temperature, voice, elasticity, pulsation, mass, etc., are observed by lightly pressing and releasing the abdominal wall by 1–2 cm with the front of the fingers. Deep palpation is intended to palpate internal organs or masses, and size, tenderness, symmetry, and motility are observed by pressing and releasing the abdominal wall 4–5 cm with the front of the fingers. Patients may feel uncomfortable when palpating the deep abdomen and complain of pain (LeBlond et al., 2015). Future devices should ultimately integrate deep palpation function. However, monitoring patient discomfort and tenderness according to palpation depth is essential to perform deep palpation. To this end, system advancement in a form that accommodates patient feedback must be considered. In the framework of this study, we found that the signals of light palpation, inspection, auscultation, and percussion could classify organs having different characteristics, and we believe that better performance will be achieved if deep palpation is implemented in the future.

Finally, we discuss the direction of this study in developing a fully telediagnostic system that can be used to classify abnormal organs in the future. The classification accuracy showed the usefulness of inferring divisions based on the characteristics of examination methods used in clinical practice by analyzing both static and dynamic information. In general, during abdominal examination for the diagnosis of abdominal disease, the abdominopelvic cavity is divided into four quadrants or nine divisions. Since the nine divisions offer more detailed anatomy than four quadrants, a more detailed diagnosis is accessible when dividing the abdominopelvic cavity into nine divisions rather than four quadrants (Floch, 2019). For example, pain is limited to the right hypochondriac region in gallbladder disease, and symptoms rarely appear in other areas. On the other hand, the possibility of the small intestine disease may be suspected if there is discomfort across various areas of the abdomen, such as the lumbar, iliac, and umbilical regions (Walker et al., 1990). However, there is no direct medical basis leading to the fact that the classification of the nine regions can distinguish abdominal diseases. Typically, clinicians learn normal percussion and palpation, and if they notice any changes, they are trained to suspect abnormalities. If the right iliac region is hard, the patient feels pain and is feverish while palpating, the patient is suspected of having appendicitis, diverticulitis, enteritis, or a ureter stone. Additionally, if the patient is female, she is suspected of having a right ovarian abscess. In another typical case, if the right hypochondriac region is hard, the patient feels pain and is feverish while palpating, the patient is suspected of having cholecystitis. In other words, the disease is suspected based on the observation of "anomalies" during the inspection, auscultation, percussion, and palpation. This procedure is very similar to the operating principle of the proposed machine learning approach. Because organs with different characteristics are located in the nine regions, each has unique characteristics. Therefore, this study expanded on the information from a preceding study that extracted these “different features” and examined the possibility of classifying the abdomen into eight divisions. Our contribution in the present study is to prove that if the prediction efficiency of these normal divisions can be achieved in a clinical environment, it could form the basis for the prediction algorithms that can be used to solve a wide range of clinical problems and provide the location of divisions.

In the future, various follow-up approaches, such as anomaly detection with unsupervised learning, will be tested. Clinical trials, including patient groups, are planned for validating models that are suitable for abnormal tissue detection, and for developing systems that can be used in wearable or remote patient-monitoring devices.

## 5. Conclusion

Basic clinical examinations are limited by their subjectivity, and the outcomes depend on the individual doctor's experience. Therefore, to detect abnormalities in the patient's abdomen in the future, we conducted a study for developing an algorithm that allows differentiating the patient's abdomen locations, as a first step. Thirty healthy adults completed a voluntary participation study. Participants were evaluated based on the inspection, auscultation, percussion, and palpation data for eight abdominal divisions. The accuracy of this regional distinction was evaluated by developing a model that responded to skin changes, sounds, accelerations, displacement, and force signals through percussion and palpation tests. The deep multi-modal learning model, which yielded a single prediction from six modality inputs, was designed for learning distinctive features of eight abdominal divisions. The subject-wise test results suggested good performance (top-1 accuracy, 65.97%; three-adjacent accuracy, 89.46%; and vertical nearest accuracy, 86.36%). Through an automatic examination of the iApp system, this study demonstrates a sophisticated classification by extracting distinct features of different abdominal divisions where different organs are located.It is expected that, in the future, this finding will serve as the basis for the development of a fully telediagnostic system that can support disease diagnosis by capturing distinct features between normal and abnormal tissues while securing patient data.

## Data Availability Statement

The datasets presented in this article are not readily available because of privacy and ethical concerns. The data that support the findings of this study are available from the corresponding author, IJ, upon reasonable request. Requests to access the datasets should be directed to In cheol Jeong, incheol1231@gmail.com.

## Ethics Statement

The studies involving human participants were reviewed and approved by Hallym University Institutional Review Board. The participants provided their written informed consent to participate in this study.

## Author Contributions

SR, CB, and IJ contributed to the study design. SR, S-CK, IJ, and D-OW conceived and designed the deep multi-modal learning architecture. SR and IJ implemented and evaluated the architecture, collected and preprocessed the data used to evaluate the designed architectures, interpreted and evaluated the results, and wrote the first version of the manuscript. J-HK collected the data. All authors have read, revised, and approved the manuscript.

## Funding

This work was supported by Institute of Information & Communications Technology Planning & Evaluation (IITP) grant funded by the Korea government (MSIT) (No. 2021-0-02068, Artificial Intelligence Innovation Hub).

## Conflict of Interest

The authors declare that the research was conducted in the absence of any commercial or financial relationships that could be construed as a potential conflict of interest.

## Publisher's Note

All claims expressed in this article are solely those of the authors and do not necessarily represent those of their affiliated organizations, or those of the publisher, the editors and the reviewers. Any product that may be evaluated in this article, or claim that may be made by its manufacturer, is not guaranteed or endorsed by the publisher.
